# Dynamic changes of CSF sTREM2 in preclinical Alzheimer’s disease: the CABLE study

**DOI:** 10.1186/s13024-020-00374-8

**Published:** 2020-04-10

**Authors:** Ling-Zhi Ma, Lan Tan, Yan-Lin Bi, Xue-Ning Shen, Wei Xu, Ya-Hui Ma, Hong-Qi Li, Qiang Dong, Jin-Tai Yu

**Affiliations:** 1 Department of Neurology, Qingdao Municipal Hospital, Qingdao University, Qingdao, China; 2 Department of Anesthesiology, Qingdao Municipal Hospital, Qingdao University, Qingdao, China; 3 Department of Neurology and Institute of Neurology, WHO Collaborating Center for Research and Training in Neurosciences, Huashan Hospital, Shanghai Medical College, Fudan University, 12th Wulumuqi Zhong Road, Shanghai, 200040 China

**Keywords:** Alzheimer’s disease, sTREM2, Inflammation, Biomarkers, Neurodegeneration

## Abstract

**Background:**

Loss of function of triggering receptor expressed on myeloid cell 2 (TREM2), a key receptor selectively expressed by microglia in the brain, contributes to the development of Alzheimer’s disease (AD). Whether TREM2 levels are pathologically altered during the preclinical phase, and whether cerebrospinal fluid (CSF) soluble TREM2 protein (sTREM2) has a relationship with major pathological processes including Aβ and tau deposition are still unclear.

**Methods:**

According to the NIA-AA criteria, 659 cognitively normal participants from the Chinese Alzheimer’s Biomarker and LifestylE (CABLE) cohort were divided into four groups, stage 0 (normal Aβ_1–42_, T-tau and P-tau), stage 1 (low Aβ_1–42_, normal T-tau and P-tau), stage 2 (low Aβ_1–42_ and high T-tau or P-tau), and suspected non-AD pathology (SNAP) (normal Aβ_1–42_ and high T-tau or P-tau), to examine changes of CSF sTREM2 in the preclinical AD. Biomarker cut-off was based on the assumption that one-third of adults with normal cognition have AD pathology.

**Results:**

The level of CSF sTREM2 in the stage 1 decreased compared with the stage 0 (*P* < 0.001), and then increased in the stage 2 (*P* = 0.008). SNAP individuals also had significantly increased CSF sTREM2 (P < 0.001). Results of multiple linear regressions also showed positive correlations of CSF sTREM2 with Aβ_1–42_ (β = 0.192, *P* < 0.001), T-tau (β = 0.215, *P* < 0.001) and P-tau (β = 0.123, *P* < 0.001).

**Conclusion:**

CSF sTREM2 levels are dynamic in preclinical AD. Aβ pathology is associated with a decrease in CSF sTREM2 in the absence of tau deposition and neurodegeneration. However, tau pathology and neurodegeneration are associated with an increase in CSF sTREM2.

## Background

Recent neuropathologic, epidemiologic and genetic studies have demonstrated that alterations of the innate immune system play a crucial role in the development of Alzheimer’s disease (AD) [1, 2]. Triggering receptor expressed on myeloid cell 2 (TREM2) is expressed abundantly by microglia in the central nervous system (CNS) and involved in key functions of microglia, including phagocytosis, cytokine release, lipid sensing, microglia proliferation, and migration [3–5]. Rare variants in the gene encoding the triggering receptor expressed on TREM2 are associated with increased risk of AD and other neurodegenerative diseases [6, 7]. TREM2 undergoes ectodomain shedding by ADAM proteases, and a soluble fragment, namely soluble TREM2 (sTREM2), is released into the extracellular space [4, 8]. sTREM2 can be detected in cerebrospinal fluid (CSF) by a method based on high sensitivity enzyme-linked immunosorbent assay (ELISA) [4, 9]. TREM2 is associated with AD and neurodegeneration, which leads us to hypothesize that CSF sTREM2 may be a marker of microglial function, and microglial response to amyloid β (Aβ), tau pathology and neurodegeneration.

Previous studies of patients with late-onset AD reported that CSF sTREM2 increased in a disease-stage-dependent manner and peaked in the early symptom phase [10, 11]. In studies of autosomal dominant mutation carriers, CSF sTREM2 increased 5 years prior to the onset of symptom onset (EYO), however, after about 5 to 10 years of Aβ pathology development [12]. Taken together, these studies suggest a complex link between CSF sTREM2 and disease progression, in which CSF sTREM2 dynamically changes with AD progression and peaks between the later asymptomatic and earlier symptomatic phases. However, these studies were only limited in clinical stage. Increased microglial activation and neuroinflammation frequently accompany the early development of Aβ and tau pathology [10]. Since TREM2 is a key protein involved in the activation of microglia, a question arises as to whether the level of sTREM2 changes pathologically during the preclinical phase, which we addressed in the current study.

Therefore, we used the biomarker-based hypothetical staging model to investigate CSF sTREM2 levels in different groups of cognitive normal divided by AD biomarkers [13, 14]. This model proposes that the asymptomatic phase of AD can be divided into 3 successive stages according to aggregated Aβ (Aβ_1–42_), aggregated tau (phosphorylated tau, P-tau) and neurodegeneration (total tau, T-tau). We also tested whether CSF sTREM2 levels were associated with the core AD CSF biomarkers Aβ_1–42_, Aβ_1–40_, T-tau and P-tau in healthy controls (HC) and preclinical AD groups [15]. We limited the study to cognitively normal individuals. Because the preclinical phase of AD is the focus of clinical trials, as it is speculated that intervention may be the most successful when treated before the initial symptom phase known as mild cognitive impairment (MCI) [16, 17].

## Methods

### CABLE database

Chinese Alzheimer’s Biomarker and LifestylE (CABLE) study is an ongoing large-scale cohort study initiated in 2017, mainly focusing on Alzheimer’s disease risk factors and biomarkers in the northern Chinese Han population. CABLE is aimed to determine the genetic and environmental modifiers of AD biomarkers and to identify lifestyle factors that may affect the risk of AD in the non-demented northern Chinese Han population, thus providing a basis for disease prevention and early diagnosis. In conjunction with the investigation, oral informed consent for future use of their CSF and blood samples for research purposes was obtained. All patients were later instructed to withdraw their permission if they changed their minds. The design of the study was approved by the Institutional Review Board of Qingdao Municipal Hospital, and the study procedure was conducted in accordance with the Declaration of Helsinki.

### Participants

All enrolled participants in the CABLE were Han Chinese aged between 40 to 90 years old. The exclusion criteria include: (1) central nervous system infection, head trauma, multiple sclerosis, neurodegenerative diseases other than AD (e.g., epilepsy, Parkinson’s Disease), or other major neurological disorders; (2) major psychological disorders; (3) severe systemic diseases (e.g., malignant tumors) that may affect CSF or blood levels of AD biomarkers including Aβ and tau; (4) family history of genetic diseases. All participants received clinical and neuropsychological assessments, biochemical testing, as well as blood and CSF sample collection. Comprehensive questionnaire, electronic medical record system, and a laboratory inspection management system were used to collect demographic information, AD risk factor profile and medical history. The diagnostic criteria of preclinical AD was defined according to the 2011 National Institute on Aging–Alzheimer’s Association (NIA-AA) workgroup reports, that is, normal cognition but abnormal AD biomarkers [14]. We first screened cognitive state of participants using the China Modified Mini-Mental State Examination (CM-MMSE) and Montreal Cognitive Assessment (MoCA). All diagnoses were evaluated by two medical doctors with extensive experience in cognitive disorders through intact performance on neuropsychological tests combined with CSF biomarkers and MRI examinations.

A total of 1000 cognitively normal participants from CABLE had available information on covariates. Sixty-nine participants were excluded who had no information about CM-MMSE, and 215 participants without available CSF sTREM2 data were excluded. Fifty-seven participants without CSF biomarkers and those had data outside the standard deviation (SD) of four times were removed. Finally, 659 participants were included in this cross-sectional analysis. Frequency histogram of age distribution of populations was shown in Figure S1.

### CSF core biomarkers and CSF sTREM2 measurements

Collection and storage of fasting lumbar CSF samples and blood samples were performed at Qingdao Municipal Hospital. CSF samples were processed immediately within 2 h after standard lumbar puncture. Each sample was centrifuged at 2000×g for 10 min, and CSF samples were separated and stored in an enzyme-free EP (Eppendorf) tube (AXYGEN; PCR-02-C) at − 80 °C for further use in the subsequent steps of this study. The samples were subjected to a maximum of two freeze-thaw cycles. The DNA in the blood samples extracted using the QIAamp®DNA Blood Mini Kit (250) was separated and stored in an enzyme-free EP tube at − 80 °C until the apolipoprotein E (*APOE*) genotyping in this study. Two specific loci related to *APOE* status (rs7412 and rs429358) were selected for genotyping with restriction fragment length polymorphism (RFLP) technology.

CSF sTREM2 and core biomarkers were measured by ELISA using the microplate reader (Thermo Scientific Multiskan MK3). CSF sTREM2 measurements were done with ELISA kits (Human TREM2 SimpleStep ELISA kit; Abcam, no. Ab224881) and CSF core biomarkers measurements were done with other ELISA kits (INNOTEST; FUJIREBIO). All ELISA measurements were performed by experienced technicians in strict accordance with the manufacturer’s instructions. They were blinded to the clinical information. The samples and standards were measured in duplicate, and the means of duplicates were used for the statistical analyses. All the antibodies and plates were from a single lot to exclude variability between batches. Moreover, the within-batch CV was < 5% and the inter-batch CV was < 15%. Furthermore, Figure S2 shows that CSF Aβ_1–42_ was reduced with *APOE ε4* when stratifying the whole cohort for *APOE* genotype and that it decreased with age. It indicated that raw data is sound with no technical problems [18].

### Statistical analysis

The scheme comprises 3 biomarkers: aggregated Aβ (Aβ_1–42_), aggregated AD-tau (P-tau) and neurodegeneration (T-tau). And each biomarker is binarized based on whether they are normal or abnormal. The observation that approximately one-third of cognitively normal older adults have AD pathology in their brains has been approved by previous amyloid imaging [18–20] and neuropathological studies [21, 22]. Similar distributions were found in studies of Asian populations [23–25]. Therefore, the cutoff values to define abnormal CSF core biomarkers were < 115.1 pg/ml (lower one-third) for Aβ_1–42_, > 40.05 pg/ml (upper one-third) for P-tau, and > 187.32 pg/ml (upper one-third) for T-tau, respectively Aggregated tau and neurodegeneration groups were merged to reduce the number of groups to be compared, which resulted in four different biomarker group combinations, including stage 0, stage 1, stage 2, and suspected non-AD pathology (SNAP). Individuals with normal measures of Aβ_1–42_, P-tau and T-tau are classified as stage 0; individuals with abnormal Aβ_1–42_ but no abnormal P-tau or T-tau are classified as stage 1; during stage 2, abnormal Aβ_1–42_, and abnormal P-tau or T-tau are evident. Individuals with evidence of aggregated tau or neurodegeneration but with normal levels of amyloid are classified as having SNAP [26]. Participants were also classified into HC (stage 0) and preclinical AD (stage 1 and stage 2) according to the Alzheimer’s *continuum* category [14, 27, 28].

CSF sTREM2 didn’t follow a normal distribution as assessed by Kolmogorov-Smirnov test (*P* = 0.200) and visual inspection of the Q-Q plot (Figure S3). Therefore, they were log-transformed to obtain a normal distribution. All the statistical analyses described in this study are performed on the log10-transformed values. We performed the analysis after excluding outliers (defined as 4 SD below or above the group mean) in order to exclude the influence of extreme values. This step removed 3 participants. Tests of intergroup differences were performed using the chi-square analysis for frequencies or one-way analysis of variance and post- hoc analysis for continuous measures. We used the Student t-test to explore whether CSF sTREM2 differs with age, gender and *APOE ε4* carrier status. The *APOE ε4* carrier status was coded as 0, 1, and 2, respectively. To test the differences in CSF sTREM2 across biomarker profiles in the biomarker framework, we applied a one-way ANCOVA followed by Bonferroni post hoc analyses. We also studied the associations between CSF sTREM2 and the CSF core biomarkers for AD, in HC (stage 0) and preclinical AD (stage 1 and stage 2) groups, with a multiple linear regression adjusted for age, gender, educational level, and *APOE ε4* carrier status. The analyses were performed in the total sample and then in subgroups stratified by age, gender and *APOE ε4* carrier status.

Statistical significance was defined as *p* < 0.05 for all analyses. Statistical analyses were performed using R (version 3.5.1) and IBM SPSS Statistics 23.

## Results

### Basic characteristics and intergroup comparisons

We studied a total of 659 participants (242 stage 0, 148 stage 1, 70 stage 2, and 199 SNAP) of the CABLE study. The demographical and clinical characteristics are described in Table 1. The resulting proportion of individuals in the hypothetical groups was comparable to that reported in the literature [26]. The study population had a female proportion of 40%, an age ranges from 40 to 90 years old (mean ± SD = 62.2 ± 10.4), education of 9.7 ± 4.4 years, and an *APOE ε4* positive percentage of 16%. As for cognitive performance, the study population had CM-MMSE of 27.8 ± 2.1 scores. There were no differences between the four groups in terms of gender, educational level, CM-MMSE score. However, individuals in stage 2 group were older and had lower cognitive composite scores than the HC group.

**Table 1:** Characteristics of participants by biomarker framework

Characteristics	Stage 0	Stage 1	Stage 2	SNAP	P
n	242	148	70	199	
Age (years)	60.81 ± 9.95	60.39 ± 10.41	64.19 ± 11.09	64.59 ± 10.28	< 0.001
Gender (F/M)	96 / 146	63 / 85	31 / 39	73 / 126	1.000
Education (years)	9.96 ± 4.39	10.17 ± 4.20	9.24 ± 4.56	9.32 ± 4.37	0.188
*APOE ε4* carriers (%)	38 (15.70)	22 (14.86)	18 (25.71)	27 (13.57)	1.000
CM-MMSE score	27.94 ± 2.25	27.92 ± 1.99	27.41 ± 2.18	27.61 ± 2.06	0.155
CSF biomarker					
Aβ_1–42_ (pg/ml)	170.41 ± 63.10	103.31 ± 7.26	102.95 ± 6.31	224.78 ± 139.64	< 0.001
Aβ_1–40_ (pg/ml)	5669.91 ± 1855.05	4567.68 ± 1906.91	7379.50 ± 2545.57	8493.87 ± 2982.58	< 0.001
T-tau (pg/ml)	135.72 ± 25.95	124.98 ± 2.55	262.85 ± 104.36	241.30 ± 88.10	< 0.001
P-tau (pg/ml)	33.18 ± 3.85	31.49 ± 3.87	46.13 ± 10.84	46.99 ± 11.37	< 0.001
Aβ_1–42_/Aβ_1–40_ (Median, IQR)	0.029 (0.024–0.037)	0.024 (0.018–0.032)	0.014 (0.011–0.018)	0.025 (0.18–0.031)	< 0.001
T-tau/Aβ_1–42_ (Median, IQR)	0.84 (0.69–1.03)	1.20 (1.02–1.36)	2.22 (1.85–3.02)	1.13 (0.90–1.58)	< 0.001
P-tau/Aβ_1–42_ (Median, IQR)	0.21 (0.17–0.25)	0.31 (0.28–0.33)	0.42 (0.39–0.51)	0.24 (0.19–0.31)	< 0.001
sTREM2 (pg/ml)	18,000.57 ± 6344.52	15,121.87 ± 6396.85	18,282.53 ± 7538.97	20,814.16 ± 6105.39	< 0.001

Categorical variables are reported as numbers and percentages; continuous variables are reported as means ± SDs

Abbreviations: *SNAP* Suspected non-Alzheimer disease pathology, *SD* Standard deviations, *F* Female, *M* Male, *APOE* Apolipoprotein E, *CM-MMSE* China Modified Mini-Mental State Examination, *CSF* Cerebrospinal fluid; Aβ_1–42_, amyloid-β_1–42_; Aβ_1–40_, amyloid-β_1–40_; T-tau, total tau; P-tau, phosphorylated tau; sTREM2, soluble TREM2

**Fig. 1:**
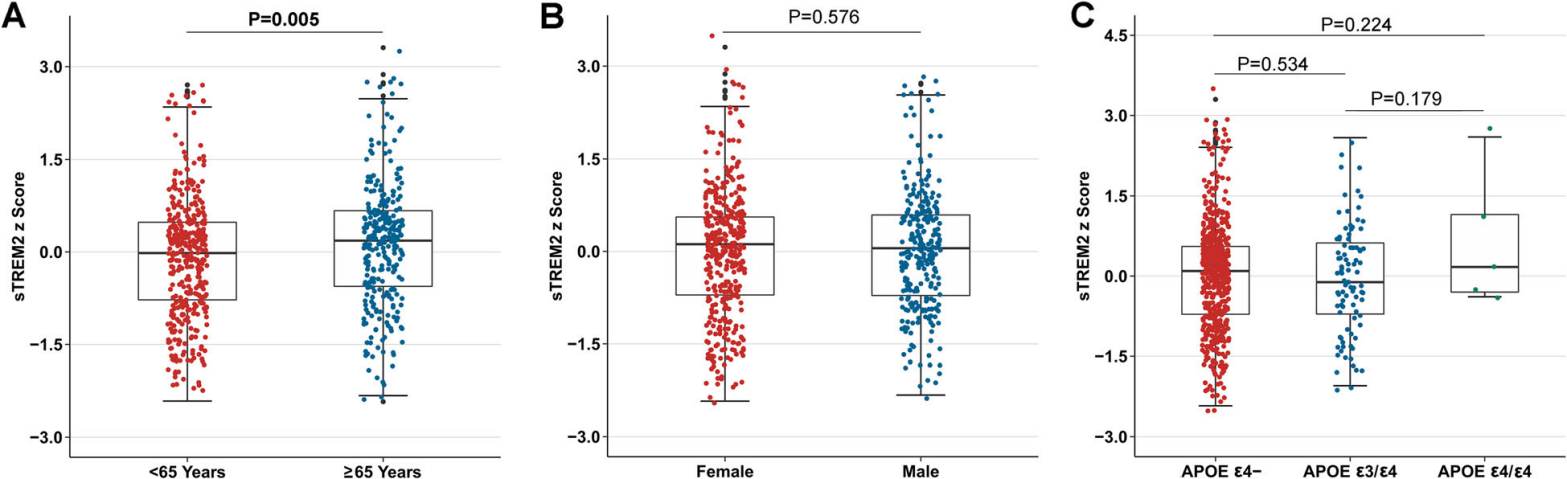
CSF sTREM2 levels are associated with age (**a**) and are independent of gender (**b**) and *APOE* carrier status (**c**). *P*-values were assessed by student’s t test. Abbreviations: CSF, cerebrospinal fluid; sTREM2, soluble TREM2; *APOE,* apolipoprotein E

Since age is the main risk factor for AD, we questioned whether CSF sTREM2 levels are related to normal aging. We found sTREM2 levels to increase with age, as demonstrated by a significant positive correlation (β = 0.004, *P* < 0.001) (Figure S4). The results indicated that CSF sTREM2 did differ significantly in different age subgroups (middle-age: 17511.5 ± 6615.2 pg/ml, *n* = 376; elder: 19193.1 ± 6769.5 pg/ml, *n* = 283; *P* = 0.005) (Fig. 1a). As expected, CSF sTREM2 concentrations were not affected by gender (male = 18,190.3 ± 6815.4 pg/ml, *n* = 263; female = 18,298.9 ± 6608.0 pg/ml, *n* = 396; *P* = 0.576) (Fig. 1b), and *APOE* carrier status (*APOE ε4* negative = 18,287.0 ± 6704.0 pg/ml, *n* = 554; *APOE ε3*/*ε4* = 17,793.5 ± 6837.8 pg/ml, *n* = 100; *APOE ε4/ε4* = 22,613.1 ± 8441.9 pg/ml, *n* = 5) (Fig. 1c).

### Difference in CSF sTREM2 level between biomarker categories

**Fig. 2:**
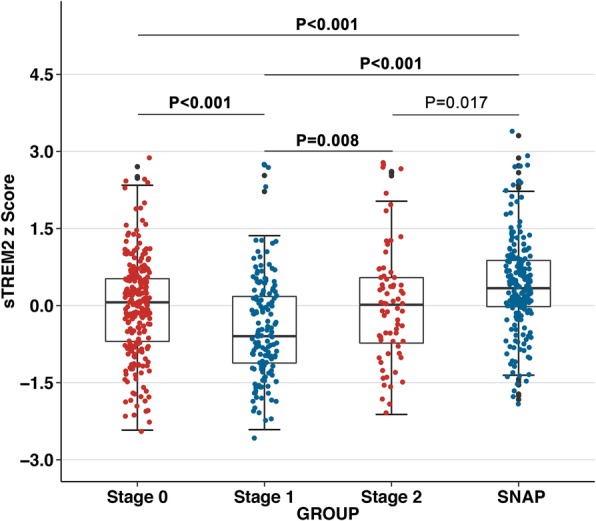
CSF sTREM2 in the biomarker classification. Scatter plots depicting the levels of CSF sTREM2 for each of the four biomarker profiles (stage 0, stage 1, stage 2, and SNAP). *P*-values were assessed by a one-way ANCOVA, and significant *P* values after Bonferroni corrected post hoc pairwise comparisons are marked. Abbreviations: CSF, cerebrospinal fluid; sTREM2, soluble TREM2; SNAP, suspected non-Alzheimer disease pathology

A representative standard curve for the CSF sTREM2 ELISA is shown in the Figure S5. A standard curve was created by plotting the average blank control subtracted absorbance value for each standard concentration (y-axis) against the target protein concentration (x-axis) of the standard. A four-parameter curve fit (4PL) was chosen to draw the best smooth curve through these points to construct the standard curve. To assess the associations of change in CSF sTREM2 with Aβ deposition and the downstream processes of the amyloid cascade (ie, tau pathology and neurodegeneration), we applied the biomarker classification framework and compared the differences in sTREM2 levels between the four stages, namely stage 0 (*n* = 242), stage 1 (*n* = 148), stage 2 (*n* = 70), and SNAP (*n* = 199). The results of one-way ANCOVA showed significant differences in CSF sTREM2 levels between the four groups. The results of the comparison between groups showed that the stage 1 group had the significantly lowest CSF sTREM2 compared to other groups (Fig. 2). Both the stage 2 (*P* = 0.008) and the SNAP (*P* < 0.001) groups had significantly increased CSF sTREM2 concentrations compared to the stage 1 group (Fig. 2). It can be inferred that the concentration of CSF sTREM2 decreases with Aβ in the pathological phase of amyloid, and then increases with T-tau or P-tau in downstream tau pathology and neurodegeneration. Changes in CSF sTREM2 occurred after alterations in markers for brain amyloidosis and neuronal injury.

Because the results suggest that age is an important influencing factor for CSF sTREM2, we conducted another ANCOVA controlling for age, and obtained the same results as follows. The stage 1 group had significantly lowest CSF sTREM2 compared to other groups (*P* < 0.001). Both the stage 2 (*P* = 0.021) and the SNAP (*P* < 0.001) groups had higher CSF sTREM2 concentrations compared to the stage 1 group. We also repeated the same analyses in groups divided by participants’ Aβ pathology (Aβ_1–42_) and tau pathological status (P-tau) or in groups divided by participants’ Aβ pathology (Aβ_1–42_) and neurodegenerative state (T-tau). The results are shown in Figure S6 and Figure S7, which are similar to the results in the former classification system.

### CSF sTREM2 levels in asymptomatic cognitively normal SNAPs

The SNAPs refer to cognitively normal individuals with abnormal tau pathology/ neurodegeneration biomarkers without the presence of significant amyloidosis [26]. We compared the SNAPs with the rest of the asymptomatic cognitively normal individuals, including HC (stage 0) and preclinical AD (stage 1and 2). We found that CSF sTREM2 levels differed among groups and particularly increased in the SNAP group compared to the HC (*P* < 0.001) and the preclinical AD (stage 1, *P* < 0.001; stage 2, *P* = 0.017) groups (Fig. 2). The results remained after controlling for age. These findings indicate that an increased CSF sTREM2 level is a response to tau pathology or neuronal injury (measured by CSF P-tau or T-tau levels), independent of amyloidosis.

### Relations between CSF sTREM2 and AD core biomarkers

**Fig. 3:**
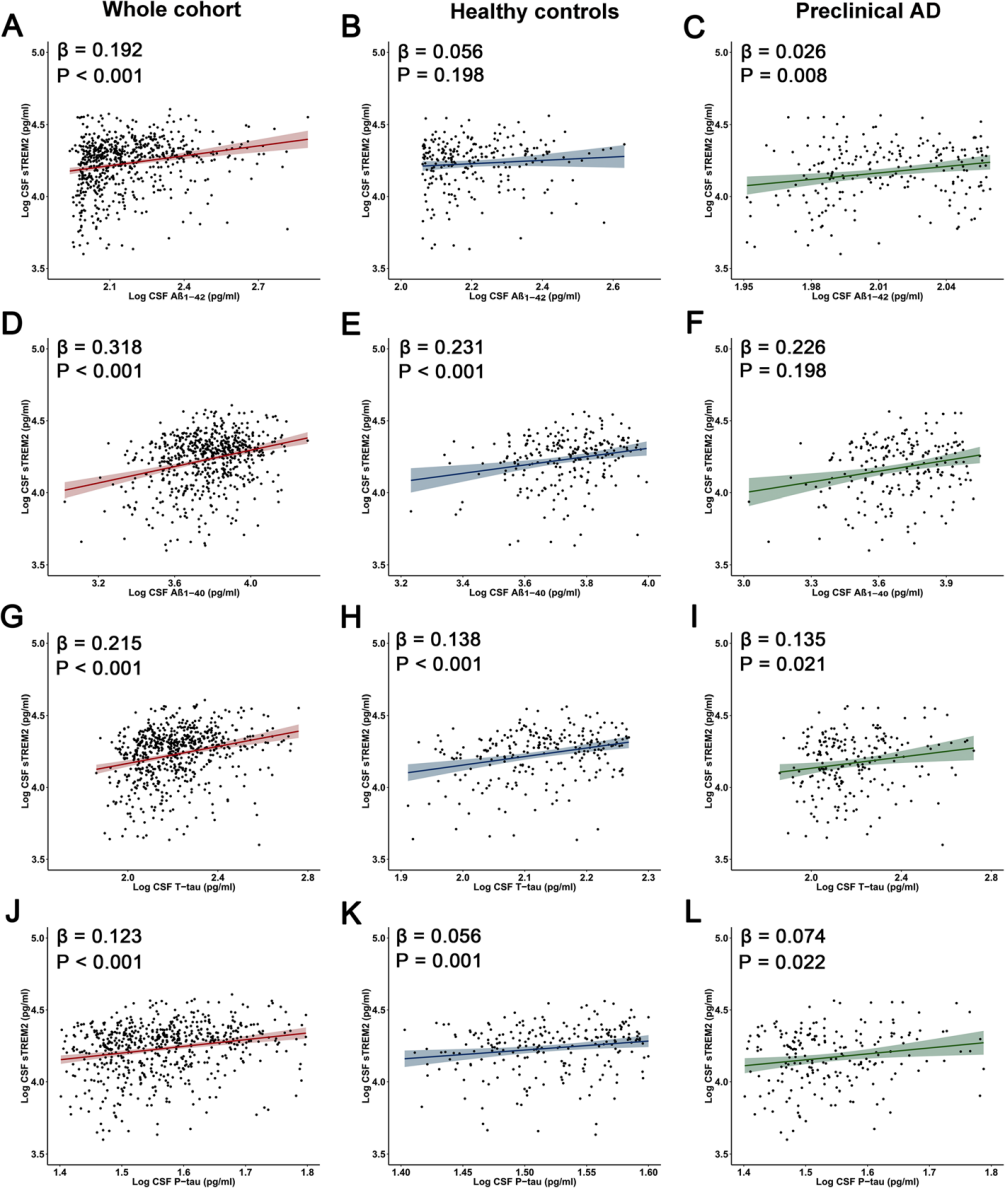
Associations of CSF sTREM2 and CSF core biomarkers. Scatter plots represent the associations of CSF sTREM2 with CSF core biomarkers: Aβ_1–42_, Aβ_1–40_, T-tau, and P-tau in three different groups (whole cohort, healthy controls, and preclinical AD). The normalized regression coefficients (β) and P values computed by multiple linear regression after adjustment for age, gender, educational level, and *APOE ε4* carrier status are shown. Abbreviations: CSF, cerebrospinal fluid; sTREM2, soluble TREM2; Aβ_1–42_, amyloid-β_1–42_; Aβ_1–40_, amyloid-β_1–40_; T-tau, total Tau; P-tau, phosphorylated Tau; AD, *APOE,* apolipoprotein E

The associations between CSF sTREM2 and core CSF markers of AD were tested in linear regression models adjusted for age, gender, educational level, and *APOE ε4* carrier status. Those individuals that fall in the category of SNAP were excluded from this analysis. In the whole sample of subjects (*n* = 659), increased CSF sTREM2 was associated with higher levels of Aβ_1–42_ (β = 0.192, *P* < 0.001) (Fig. 3a) and Aβ_1–40_ (β = 0.318, *P <* 0.001) (Fig. 3d), as well as higher levels of T-tau (β = 0.215, *P* < 0.001) (Fig. 3g) and P-tau (β = 0.123, *P* < 0.001) (Fig. 3f). The results of the subgroup analysis showed that the relationship between sTREM2 and CSF biomarkers remained unchanged regardless of age, gender, and *APOE ε4* carrier status (Table S2). There was a positive association between CSF sTREM2 and T-tau or P-tau in HC (*n* = 242) and preclinical AD groups (*n* = 218) (Fig. 3h–i, Fig. 3k-l), whereas no significant association was found between CSF sTREM2 and Aβ_1–42_ in HC group (β = 0.056, *P* = 0.198) (Fig. 3b). We then examined ratios of CSF amyloid and tau biomarkers, and found no associations of CSF sTREM2 with CSF Aβ_1–42_/Aβ_1–40_, T-tau/Aβ_1–42_ and P-tau/Aβ_1–42_ (Table S1). Outliers were excluded in our analyses, but we obtained similar results when those were included. These finding indicate that higher CSF sTREM2 correlates with higher levels of markers for neuronal injury and tau pathology, suggesting an early response of TREM2 to the first symptoms of neurodegeneration.

## Discussion

In the present study, we combined biomarker-based classification with clinical stages to assess changes in the microglial-activity marker CSF sTREM2 within the early phases of AD. The application of this classification system enabled us to explore the associations of microglial inflammatory response with AD pathophysiology (including Aβ pathology, tau pathology, and neurodegeneration). Our study extends previous evidence by showing that CSF sTREM2 levels did change dynamically with pathological processes. The simple Aβ pathology (defined as low CSF Aβ_1–42_) is associated with a decrease in CSF sTREM2, while tau pathology or neurodegeneration is associated with elevated CSF sTREM2. This seems to confirm the potential role of microglial inflammatory response in the pathogenesis of AD. Our data provide support for this hypothesis: Aβ deposition occurs independently of the inflammatory process, but the type and extent of the inflammatory response to Aβ deposition in the brain may trigger or affect subsequent neurodegeneration [29]. Another piece of evidence is that immunotherapy against amyloid can reduce downstream neurodegeneration, a process that may be mediated by changes in microglial activation [29].

Consistent with previous studies, the findings of the present study indicate that higher CSF sTREM2 concentrations are linked to tau pathology or neurodegeneration (defined as elevated CSF P-tau or T-tau) even in the absence of Aβ pathology, as indicated by the differences between HC and SNAP individuals [12, 30]. This suggests that an increased inflammatory response and involvement of reactive microgliosis are related to neurodegeneration. Without Aβ pathology, increased sTREM2 seems to be a consequence rather than a cause of neurodegeneration, which is in line with reports that the TREM2 receptor mediated the phagocytosis of apoptotic neurons by microglia [31]. In addition, the SNAP category is noteworthy because it is the heterogeneous group most likely to exhibit non-AD-related neurodegeneration. Future studies should address changes in CSF sTREM2 in neurodegenerative diseases other than AD.

Exploring relations between CSF biomarkers may provide better pathogenic understanding by hinting to simultaneously ongoing processes in the brain. Our results are consistent with previous research studies [10–12, 32]. The level of CSF sTREM2 was positively correlated with the core CSF biomarkers Aβ_1–42_, Aβ_1–40_, T-tau and P-tau on the entire data set and in preclinical AD, which further suggests an early involvement of reactive microgliosis. In HC subjects, the correlation with Aβ_1–42_ disappeared, while the correlations of sTREM2 with T-tau and P-tau remained. These findings suggest that changes in CSF sTREM2 may indeed be associated with neuronal injury. It is also suggested that the increase in CSF sTREM2 during normal aging may be a protective response to mild neuronal injury. Furthermore, we showed that sTREM2 did not seem to depend on the *APOE ε4* genotype, as there was no difference in CSF sTREM2 concentration between *APOE ε4* carriers and non-carriers; sTREM2 also did not depend on gender because there was no difference in CSF sTREM2 concentration between males and females.

The underlying mechanism of dynamic changes in CSF sTREM2 remains to be investigated throughout the disease. The mechanism underlying the reduction of CSF sTREM2 in the early stage of Aβ pathology is still unclear. One explanation is that microglia initially form a barrier around the plaque, and sTREM2 released by microglia remains in the plaque until the barrier fails, followed by neural injury [33, 34]. Another explanation is that individuals with low function of TREM2 may have early amyloidogenic acceleration, so the level of sTREM2 in the preclinical AD stage 1 is lower [35]. Increased sTREM2 is caused by microglial activation, which is evidenced by increased TREM2 expression both in elderly AD mouse models and in human AD brains [36, 37]. In addition, detailed transcriptomics studies investigated microglia in AD and neurodegenerative mouse models, suggesting that TREM2 is up-regulated in disease-associated microglia (DAM) [38–40]. We found positive correlations of CSF sTREM2 with T-tau and P-tau in the control group and preclinical group, which was consistent with previous studies [10, 12]. Since neuroinflammation is a feature of aging, [41, 42], it cannot be ruled out that correlation of CSF sTREM2 levels with T-tau/P-tau in HC group could thus be an indirect effect of the age-dependent increase in T-tau/P-tau. Another possibility is that studies have suggested that the increase in CSF tau in AD is due to increased tau production (ie, synthesis and release) rather than decreased fractional clearance. Under normal physiological conditions, soluble tau and CSF tau in the brain are in equilibrium. However, mechanisms such as inflammation may increase the production or release of tau into the extracellular space, and over time, the concentration of soluble extracellular tau will increase [43]. CSF sTREM2 will be an attractive biomarker candidate to track AD progression and it can serve as a potential outcome parameter for future clinical trials of TREM2 and neuroinflammation.

The strength of the current study is a large sample of well-characterized subjects with biomarker evidence of AD pathology. We used an AD biomarker classification system in line with NIA-AA research diagnostic guidelines for AD. This allowed us to study patients in the preclinical stages of AD. Previous studies mainly focused on AD patients, with a few participants in the preclinical phase, and had no biomarker classification [12, 44]. However, our study also has some limitations. First, we did not screen subjects for possible TREM2 mutations. However, the possibility of TREM2 mutations in current patient samples is highly unlikely to affect our results, as the prevalence of TREM2 mutations in the general population or AD patients is low [7, 45]. Second, this is a cross-sectional study that limits any conclusions about disease progression. Therefore, results should be replicated in subjects with longitudinal data to analyze whether CSF sTREM2 levels are associated with disease progression.

## Conclusion

In conclusion, this study is based on an independent cohort. The results indicate that CSF sTREM2 is dynamic in preclinical AD. Aβ pathology is associated with a decrease in CSF sTREM2 in the absence of tau deposition and neurodegeneration, whereas tau pathology and neurodegeneration is associated with an increase in CSF sTREM2. Future studies should use biomarkers to further categorize the preclinical AD stage and explore the biological mechanisms underlying the dynamic CSF sTREM2 changes.

## Supplementary information

**Additional file 1: Figure S1.** Histogram of age distribution of CABLE populations. **Figure S2.** Associations of age and the presence or absence of *APOE ε*4 for CSF measures of Aβ_1–42_. **Figure S3.** The Quantile-Quantile plot of CSF sTREM2. **Figure S4.** Association of CSF sTREM2 and age. **Figure S5.** One of the standard curves for CSF sTREM2. **Figure S6.**CSF sTREM2 in groups defined only by CSF Aβ_1–42_ and P-tau. **Figure S7.** CSF sTREM2 in groups defined only by CSF Aβ_1–42_ and T-tau. **Table S1.** Association of CSF sTREM2 and CSF core biomarkers. **Table S2.** Subgroup analyses of whole participants by age, gender, and *APOE ε4* carrier status.

## Data Availability

The data that support the findings of this study are available from the corresponding author but restrictions apply to the availability of these data, which were used under license for the current study, and so are not publicly available. Data are however available with permission of the corresponding author.

## Abbreviations

AD: Alzheimer’s disease
TREM2: Triggering receptor expressed on myeloid cell 2
CNS: Central nervous system
sTREM2: Soluble TREM2
CSF: Cerebrospinal fluid
ELISA: Enzyme-linked immunosorbent assay
Aβ: Amyloid β
Aβ_1–42_: Amyloid-β_1–42_
P-tau: Phosphorylated tau
T-tau: Total tau
HC: Healthy control
Aβ_1–40_: Amyloid-β_1–40_
MCI: Mild cognitive impairment
CABLE: Chinese Alzheimer’s Biomarker and Lifestyle
NIA-AA: Aging–Alzheimer’s Association
CM-MMSE: China Modified Mini-Mental State Examination
SD: Standard deviation
*APOE*: Apolipoprotein E
RFLP: Restriction fragment length polymorphism
SNAP: Suspected non-AD pathology
