# Supplementary material for: Dynamic changes of CSF sTREM2 in preclinical Alzheimer’s disease: the CABLE study
Source: Mol Neurodegener. 2020 Apr 10;15:25. doi: 10.1186/s13024-020-00374-8 (PMC7149923; doi:10.1186/s13024-020-00374-8)
Supplement: Supplementary file 1 — Additional file 1: Figure S1. Histogram of age distribution of CABLE populations. Figure S2. Associations of age and the presence or absence of APOE ε4 for CSF measures of Aβ1–42. Figure S3. The Quantile-Quantile plot of CSF sTREM2. Figure S4. Association of CSF sTREM2 and age. Figure S5. One of the standard curves for CSF sTREM2. Figure S6.CSF sTREM2 in groups defined only by CSF Aβ1–42 and P-tau. Figure S7. CSF sTREM2 in groups defined only by CSF Aβ1–42 and T-tau. Table S1. Association of CSF sTREM2 and CSF core biomarkers. Table S2. Subgroup analyses of whole participants by age, gender, and APOE ε4 carrier status. [file 13024_2020_374_MOESM1_ESM.doc]

**Figure S1.** **Histogram of age distribution of CABLE populations.**

**
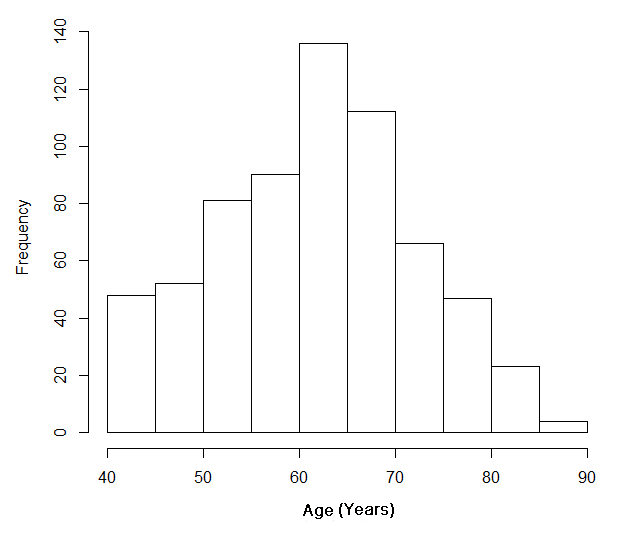
**

Frequency histogram of age distribution of CABLE populations. X-axis represents age Y-axis represents frequency.

Abbreviations: CABLE, Chinese Alzheimer's Biomarker and LifestylE.

**Figure S2. Associations of age and the presence or absence of *APOE*** ***ε*4 for CSF measures of Aβ_1-42_.**

**
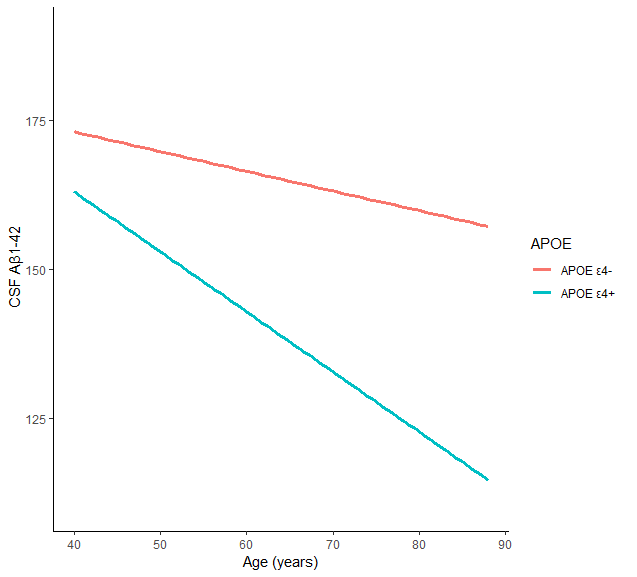
**

Participants with at least one *APOE* ε*4* allele have lower CSF Aβ_1-42_ values with age than those without *APOE* ε*4* have higher CSF Aβ_1-42_ values with age.

Abbreviations: CSF, cerebrospinal fluid; Aβ_1-42_, amyloid-β_1–42_.

**Figure S3. The Quantile-Quantile plot of CSF sTREM2.**

**
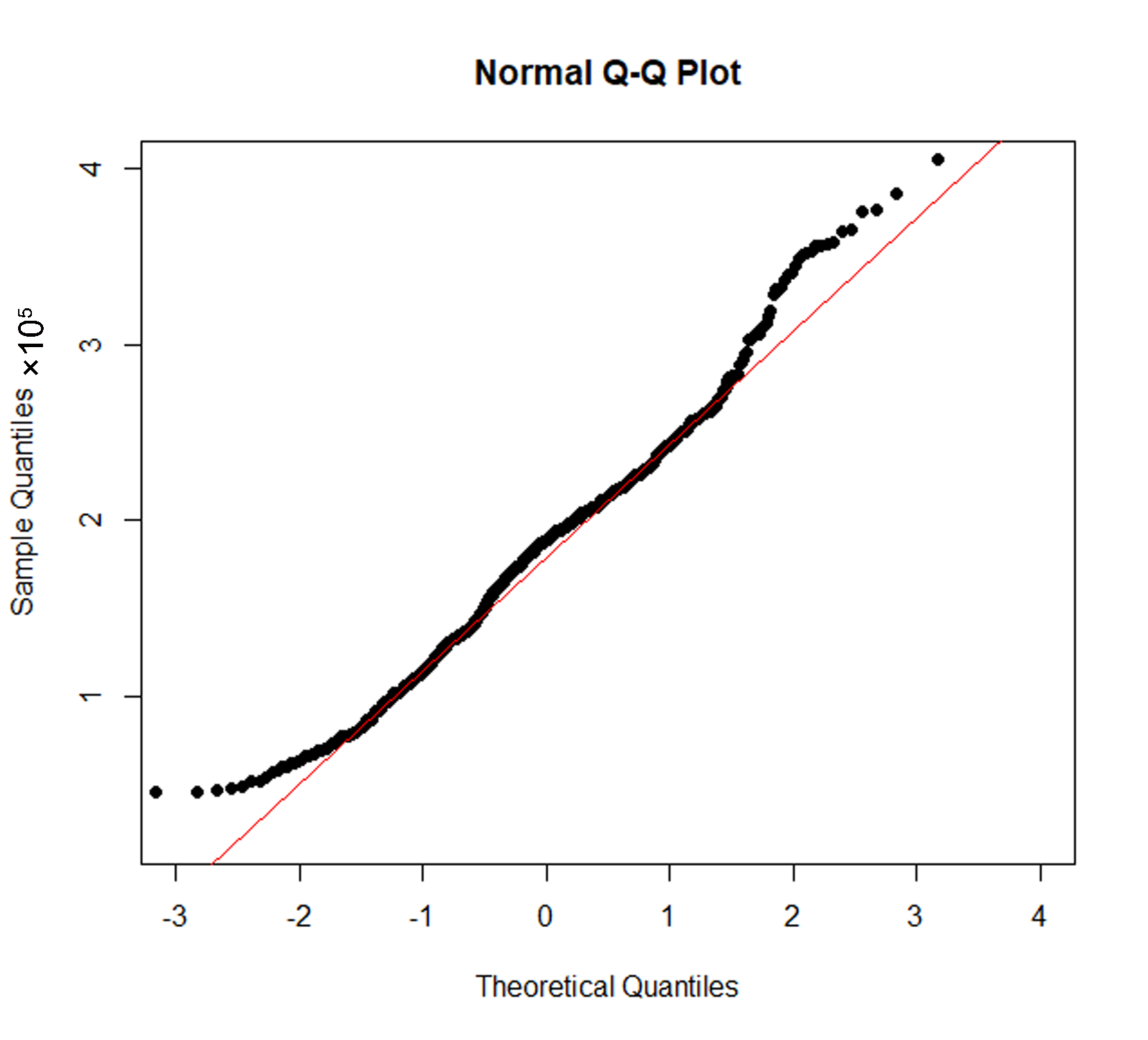
**

Sample quantiles were plotted against those theoretical quantiles under the null hypothesis (x-axis). QQ graph indicates that the data of CSF sTREM2 does not conform to the normal distribution.

Abbreviations: CSF, cerebrospinal fluid; sTREM2, soluble TREM2.

**Figure S4. Association of** **CSF sTREM2 and age.**

**
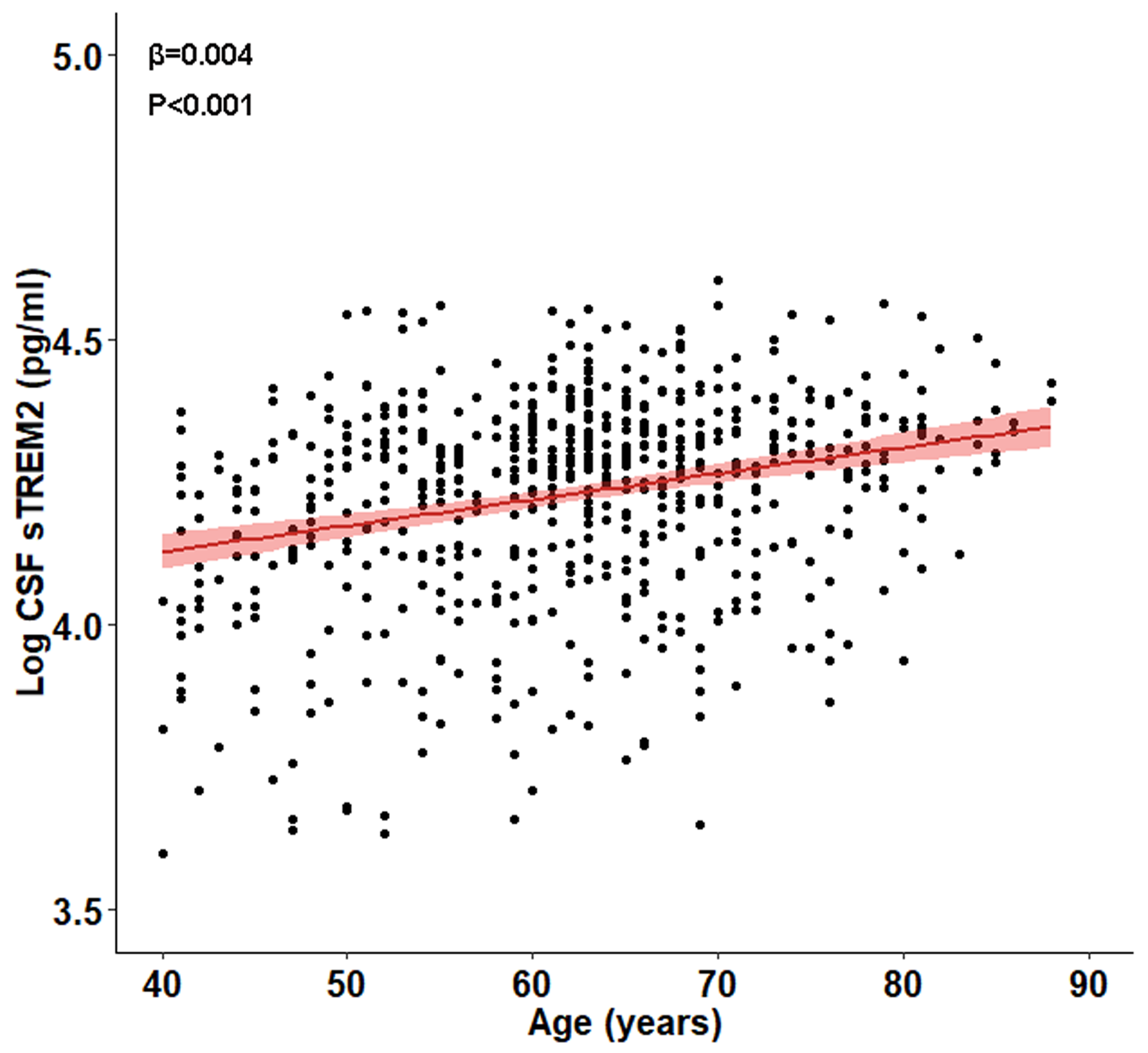
**

Associations of CSF sTREM2 and age. The normalized regression coefficients (β) and P values computed by multiple linear regression.

Abbreviations: CSF, cerebrospinal fluid; sTREM2, soluble TREM2.

**Figure S5. One of the standard curves for CSF sTREM2.**

**
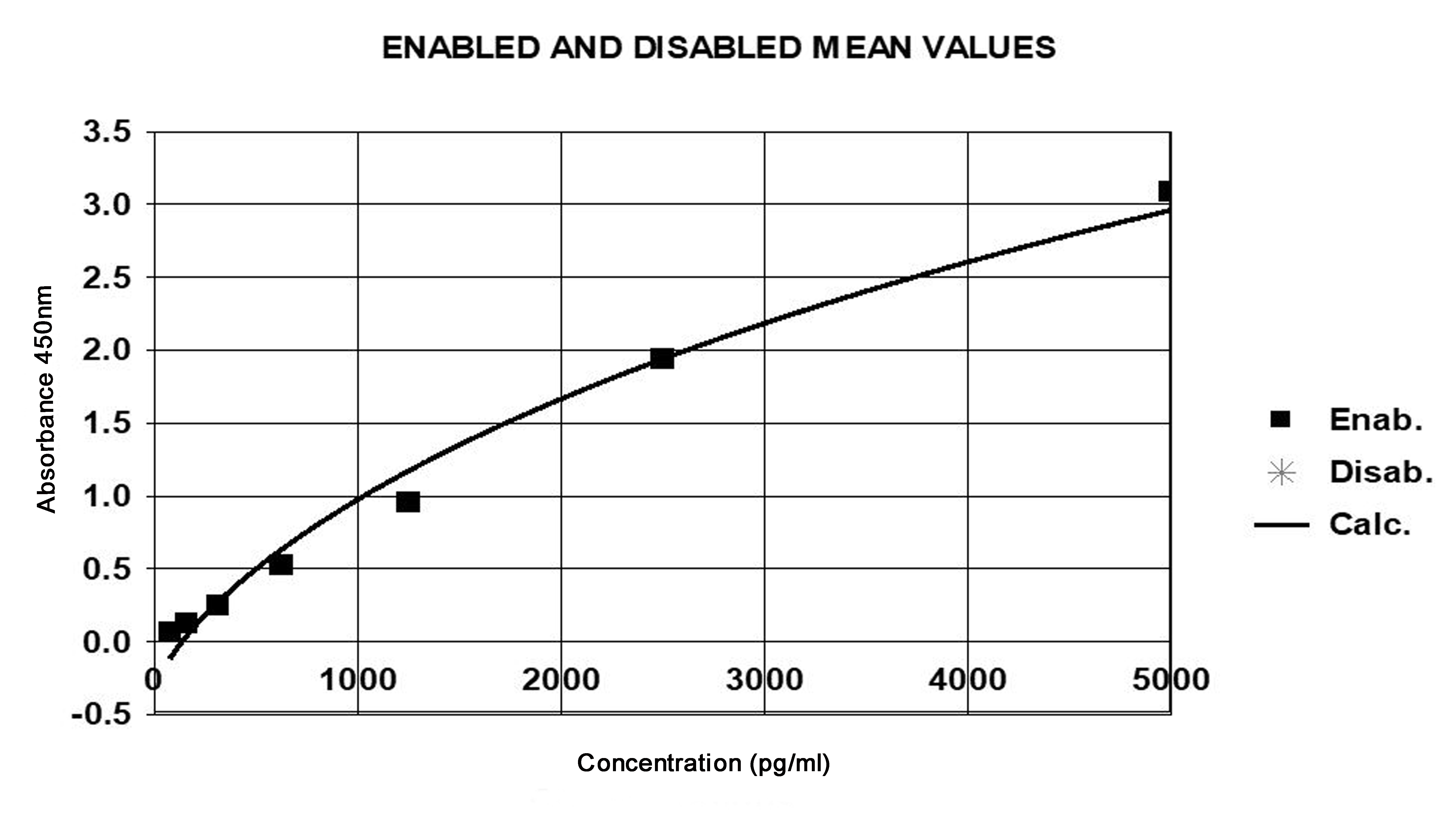
**

Create a standard curve by plotting the average blank control subtracted absorbance value for each standard concentration (y-axis) against the target protein concentration (x-axis) of the standard. A four-parameter curve fit (4PL) was chosen to draw the best smooth curve through these points to construct the standard curve.

**Figure S6.CSF sTREM2 in groups defined only by CSF Aβ_1-42_ and P-tau.**


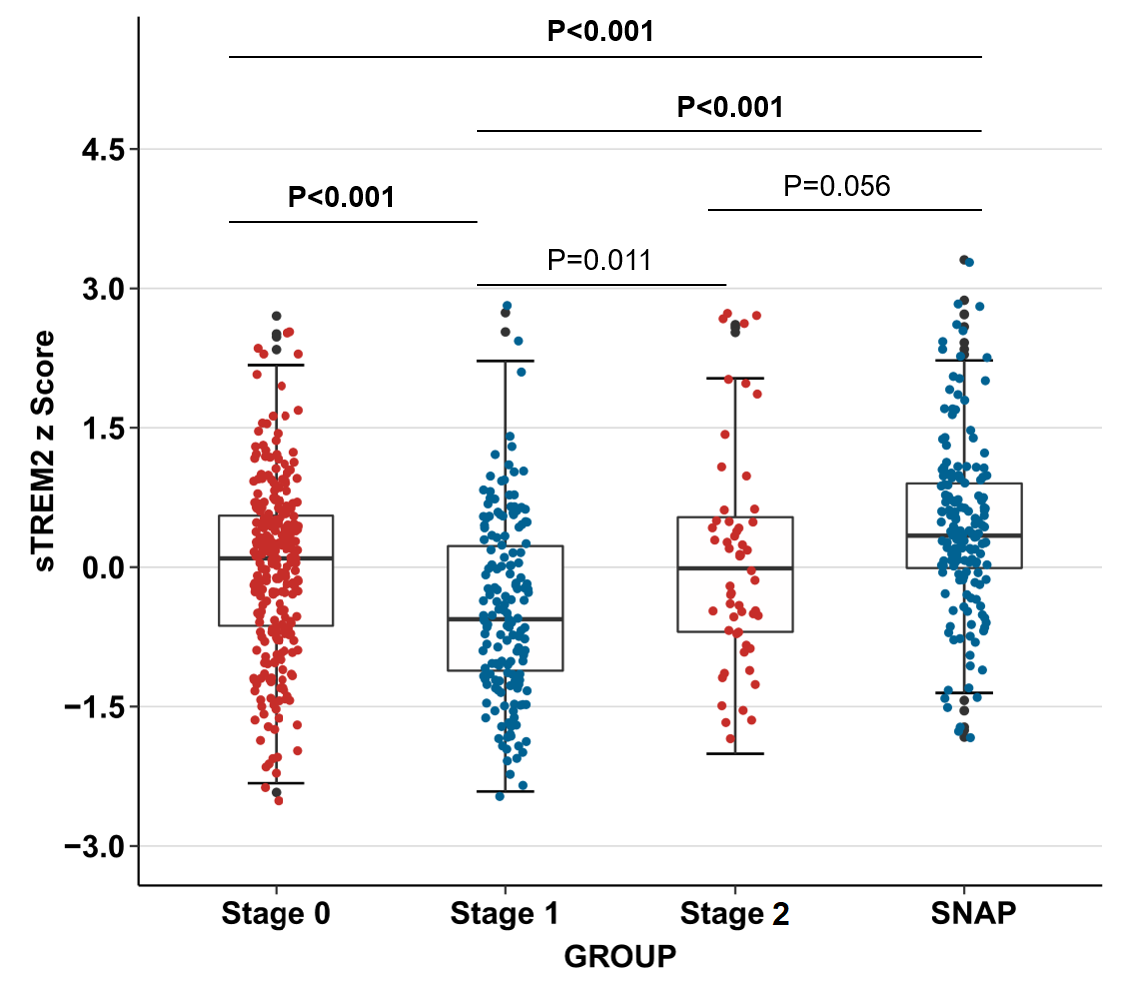


CSF sTREM2 in groups defined by CSF Aβ_1-42_ and P-tau. Scatter plot depicting the levels of CSF sTREM2 for each of the four biomarker profiles, as defined by the CSF Aβ_1-42_ and P-tau. Participants are classified based on their Aβ pathology and tau pathology status. P-values were assessed by a one-way ANCOVA, significant P values after Bonferroni corrected post hoc pairwise comparisons are marked.

Abbreviations: CSF, cerebrospinal fluid; sTREM2, soluble TREM2; Aβ_1-42_, amyloid-β_1–42_; P-tau, phosphorylated tau.

**Figure S7. CSF sTREM2 in groups defined only by CSF Aβ_1-42_ and T-tau.**

**
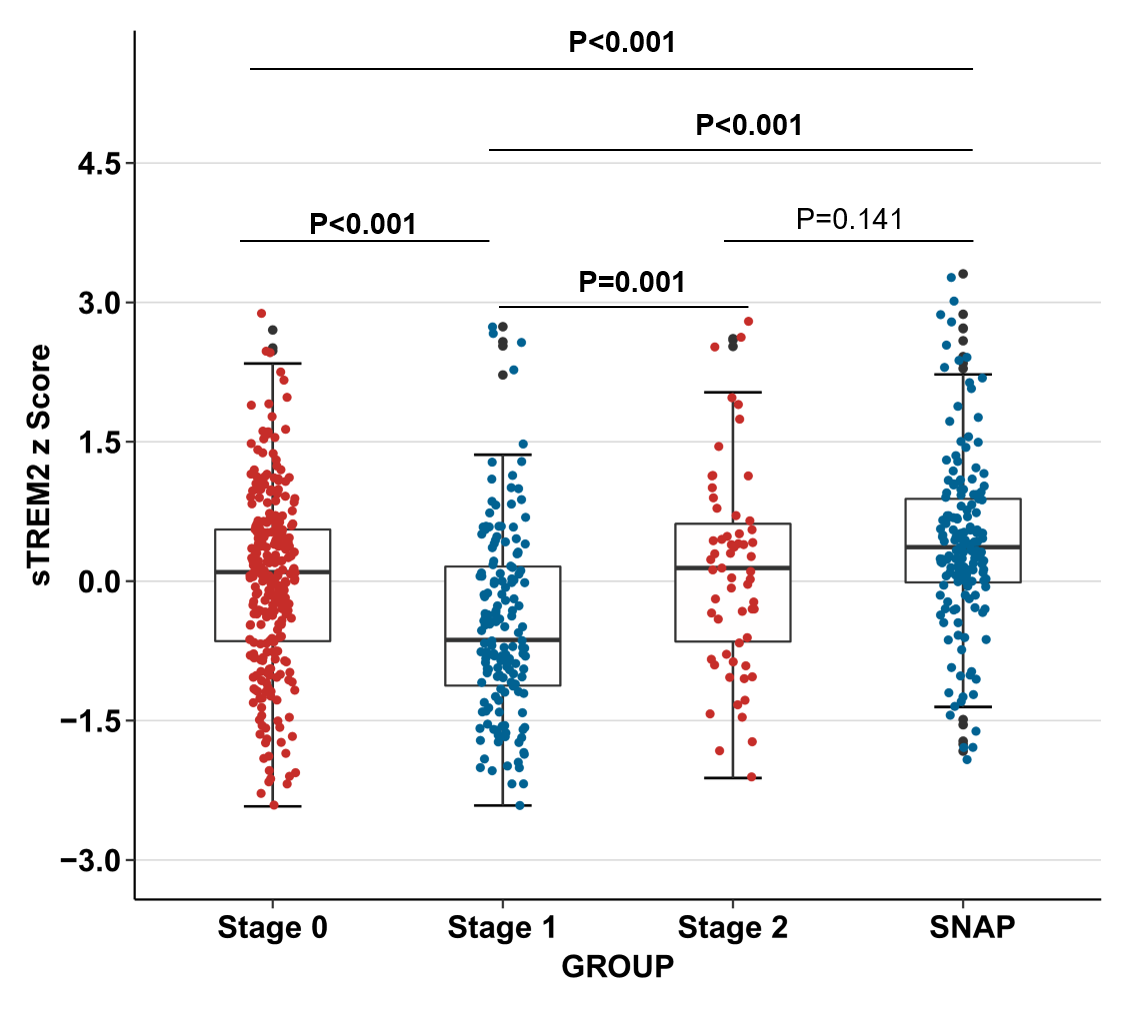
**

CSF sTREM2 in groups defined by CSF Aβ_1-42_ and T-tau. Scatter plot depicting the levels of CSF sTREM2 for each of the four biomarker profiles, as defined by the CSF Aβ_1-42_ and T-tau. Participants are classified based on their Aβ pathology and neurodegeneration biomarker status. P-values were assessed by a one-way ANCOVA, significant P values after Bonferroni corrected post hoc pairwise comparisons are marked.

Abbreviations: CSF, cerebrospinal fluid; sTREM2, soluble TREM2; Aβ_1-42_, amyloid-β_1–42_; T-tau, total tau.

**Table S1. Association of CSF sTREM2 and CSF core biomarkers.**

| **CSF biomarker** | **Total sample** | |  | **HC** | |  | **Preclinical AD** | |
| --- | --- | --- | --- | --- | --- | --- | --- | --- |
|  | **β** | **P** |  | **β** | **P** |  | **β** | **P** |
| Aβ_1-42_ | 0.192 | **<0.001** |  | 0.056 | 0.198 |  | 0.026 | **0.008** |
| Aβ_1-40_ | 0.318 | **<0.001** |  | 0.231 | **<0.001** |  | 0.226 | **<0.001** |
| T-tau | 0.215 | **<0.001** |  | 0.138 | **<0.001** |  | 0.135 | **0.021** |
| P-tau | 0.123 | **<0.001** |  | 0.056 | **0.001** |  | 0.074 | **0.022** |
| Aβ_1-42_/Aβ_1-40_ | -0.001 | 0.964 |  | -0.023 | 0.067 |  | -0.030 | **0.002** |
| T-tau/Aβ_1-42_ | 0.100 | 0.615 |  | 0.040 | 0.076 |  | 0.054 | 0.069 |
| P-tau/Aβ_1-42_ | -0.006 | 0.591 |  | 0.007 | 0.623 |  | 0.027 | 0.092 |

Adjusted P values are listed in the table.

Adjusted for age, gender, educational level, and *APOE ε4* carrier status.

**Abbreviations:** HC, healthy control; AD, Alzheimer's disease; CSF, cerebrospinal fluid; *APOE,* apolipoprotein E; Aβ_1-42_, amyloid-β_1–42_; Aβ_1-40_, amyloid-β_1–40_; T-tau, total Tau; P-tau, phosphorylated Tau; sTREM2, soluble TREM2.

**Table S2. Subgroup analyses of whole participants by age, gender, and *APOE ε4* carrier status.**

| **CSF biomarker** | **Age** | | | | |  | **Gender** | | | | |  | ***APOE ε4* carrier status** | | | | |
| --- | --- | --- | --- | --- | --- | --- | --- | --- | --- | --- | --- | --- | --- | --- | --- | --- | --- |
|  | **<65 years** | |  | **≥65 years** | |  | **Female** | |  | **Male** | |  | ***APOE ε4*-** | |  | ***APOE ε4*+** | |
|  | **β** | **P*** |  | **β** | **P*** |  | **β** | **P†** |  | **β** | **P†** |  | **β** | **P‡** |  | **β** | **P‡** |
| Aβ_1-42_ | 0.187 | **<0.001** |  | 0.188 | **<0.001** |  | 0.231 | **<0.001** |  | 0.171 | **<0.001** |  | 0.198 | **<0.001** |  | 0.172 | **0.023** |
| Aβ_1-40_ | 0.336 | **<0.001** |  | 0.348 | **<0.001** |  | 0.217 | **<0.001** |  | 0.372 | **<0.001** |  | 0.336 | **<0.001** |  | 0.208 | **0.029** |
| T-tau | 0.255 | **<0.001** |  | 0.291 | **<0.001** |  | 0.198 | **<0.001** |  | 0.224 | **<0.001** |  | 0.214 | **<0.001** |  | 0.216 | **0.009** |
| P-tau | 0.145 | **<0.001** |  | 0.155 | **<0.001** |  | 0.140 | **<0.001** |  | 0.115 | **<0.001** |  | 0.127 | **<0.001** |  | 0.094 | 0.062 |
| Aβ_1-42_/ Aβ_1-40_ | -0.004 | 0.625 |  | -0.007 | 0.587 |  | 0.026 | 0.102 |  | -0.014 | 0.210 |  | -0.002 | 0.870 |  | 0.013 | 0.533 |
| T-tau/ Aβ_1-42_ | 0.031 | 0.107 |  | 0.046 | 0.147 |  | -0.018 | 0.591 |  | 0.025 | 0.292 |  | 0.006 | 0.778 |  | 0.020 | 0.695 |
| P-tau/ Aβ_1-42_ | 0.005 | 0.657 |  | 0.008 | 0.695 |  | -0.012 | 0.594 |  | -0.004 | 0.795 |  | -0.007 | 0.577 |  | -0.013 | 0.691 |

Adjusted P values are listed in the table.

*Adjusted for gender, educational level, and *APOE ε4* carrier status.

**†** Adjusted for age, educational level, and *APOE ε4* carrier status.

**‡** Adjusted for age, gender, educational level.

**Abbreviations:** CSF, cerebrospinal fluid; *APOE*, apolipoprotein E; Aβ_1-42_, amyloid-β_1–42_; Aβ_1-40_, amyloid-β_1–40_; T-tau, total Tau; P-tau, phosphorylated Tau; sTREM2, soluble TREM2
